# Goos-Hänchen shift of partially coherent light fields in epsilon-near-zero metamaterials

**DOI:** 10.1038/srep26504

**Published:** 2016-05-23

**Authors:** Ziauddin, You-Lin Chuang, Sajid Qamar, Ray-Kuang Lee

**Affiliations:** 1 Institute of Photonics and Technologies, National Tsing-Hua University, Hsinchu 300, Taiwan; 2 Department of Physics, COMSATS institute of information technology, Islamabad, Pakistan

## Abstract

The Goos-Hänchen (GH) shifts in the reflected light are investigated both for *p* and *s* polarized partial coherent light beams incident on epsilon-near-zero (ENZ) metamaterials. In contrary to the coherent counterparts, the magnitude of GH shift becomes non-zero for *p* polarized partial coherent light beam; while GH shift can be relatively large with a small degree of spatial coherence for *s* polarized partial coherent beam. Dependence on the beam width and the permittivity of ENZ metamaterials is also revealed for partial coherent light fields. Our results on the GH shifts provide a direction on the applications for partial coherent light sources in ENZ metamaterials.

Goos-Hänchen (GH) shift reveals the discrepancy between geometric optics and wave optics, when a light beam undergoes a small lateral displacement from the interface of two media^1^. There are a variety of interesting applications on the GH shift. For example, in optical heterodyne sensors, GH shift is used to measure beam angle, refractive index, displacement, temperature, and film thickness^2^. The GH shift has also been used to characterize the permittivity and permeability of different materials in optical microscopy and lithography^3^,^4^.

In the past decades, the GH shift has been studied extensively in various interfaces including absorptive dielectrics^5^,^6^,^7^, metals^8^,^9^ and photonics^10^,^11^. In addition to these natural materials, recently, a lot of interest has been emerged for zero index metamaterials (ZIMs) due to its stimulating electromagnetic features^12^,^13^,^14^,^15^. Even though metamaterials cannot be found in nature, they could be manufactured very easily through plasmonic materials^16^ or doped semiconductors^17^. In particular, the GH shift in the reflected light is studied for *s* or *p* polarized light incident on epsilon-zero-index (ENZ) metamaterials^18^. Here, ENZ metamaterial is a kind of ZIMs. It is found that when a linear polarized light beam incident from air onto ENZ metamaterials, there is no GH shift for *p* polarized light beam; while a constant GH shift remains for *s* polarized light beam^18^.

As the coherent effect plays a key role in every investigation on wave phenomena^19^,^20^,^21^,^22^,^23^,^24^, the influence from partial coherent light fields on the GH shifts raised a debate^25^,^26^,^27^,^28^. This contradiction has been revealed thoroughly by Zubairy and coworkers for the first time^29^, then by Ziauddin and coworkers in considering a partial coherent light beam incident on a medium^30^. With a partial coherent light field, a counter-intuitive result on the GH shift with a strong dependence on spatial coherence is revealed^29^,^30^.

In this Report, we consider a *p* or *s* polarized partial coherent light field incident on ENZ metamaterials and study the GH shift from the reflection. We show the influence of spatial coherence and beam width of partial coherent light on the GH shift for both *p* and *s* polarized lights. In contrary to the coherent counterparts, a totally different scenario for GH shift happens with partially coherent light fields. For *p* polarized partial coherent light beam, the magnitude of GH shift becomes non-zero with a small degree of spatial coherence; while for *s* polarized partial coherent beam, a relatively large GH shift can also be achieved in low spatial coherence. We also reveal the dependence on the beam width and the permittivity of ENZ metamaterials, both for *p* and *s* partial coherent light fields. By combining ENZ metamaterials and partial coherent light, our results provide a direction for highly precise measurement on GH shifts.

## Results

### Fundamental concept

**Figure 1:**
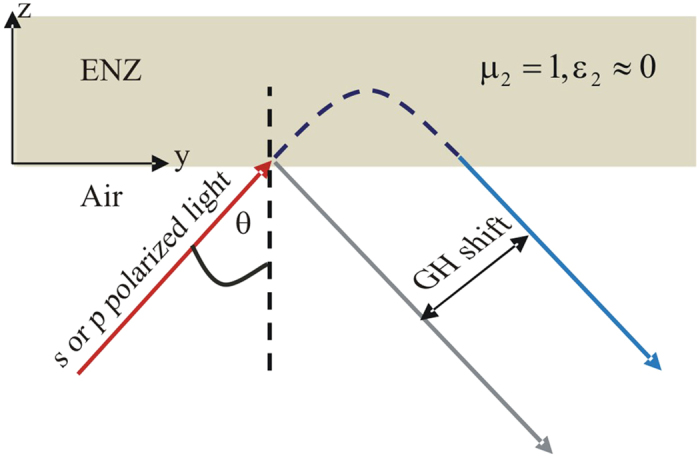
Schematics of a partial coherent light incident on ENZ metamaterials, with an angle *θ* to the *z* -axis.

We consider a partial coherent light field (*p* or *s* polarized) that incidents from air on ENZ metamaterials, which have the permeability *μ*_2_ = 1 and permittivity*ε*_2_ ≈ 0, respectively. The partial coherent light makes an angle *θ* with respect to the *z*-axis, as depicted in Fig. 1. Based on Mercer’s mode expansion, the electric field for *m* th-order mode in a partial coherent light at *z *= 0 can have the form^30^,^31^


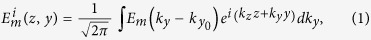


where


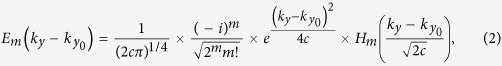


being the angular spectrum that can be calculated using a Gaussian Schell-model (GSM) beam. For the normalized eigen-function in GSM light beams, one has^31^


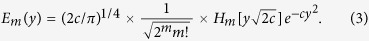


The corresponding angular spectrum *E*_*m*_(*k*_*y*_) can be calculated by taking Fourier transform of Eq. (3). Here, *E*_*m*_(*k*_*y*_) is replaced by 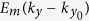, and *k*_*y*_ is the y-component of the wave vector *k*, 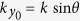, and *θ* is the incident angle. In Eqs (2, 3), *H*_*m*_ is the Hermite polynomials and 
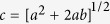
, which can be calculated using the eigenvalues 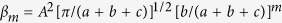 of GSM beams with 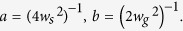 Furthermore, *w*_*g*_ and *w*_*s*_ are the spectral coherence and beam width of partial coherent light, respectively. We have the expression for 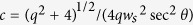 with 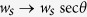 and 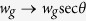. To describe a partially coherent light field, the parameter *q* = *w*_*g*_/*w*_*s*_ measures the degree of the coherence in a GSM beam, which is also denoted as the spatial coherence.

For the reflected partial coherent light in *m* th-order mode, we have^29^,^30^


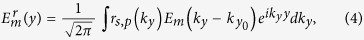


where 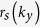 and 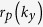
are the reflection coefficients.

For *s* and *p* polarized light, the corresponding reflection coefficients can be calculated as^18^:


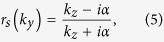



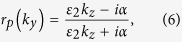


with 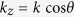 and 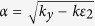. Then, the compact expression for GH shifts in the reflected light in the *m* th-order mode can be derived analytically by the following expression^29^


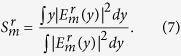


### Effect of spatial coherence on GH shift

It has been reported earlier for a coherent light beam incident on lossless ENZ metamaterials, there is no GH shift for *p* polarized light; while for *s* polarized light the GH shift is a constant value for different incident angles^18^. In the following, we proceed the study on the GH shifts in the reflected light for *p* or *s* polarization, but with partially coherent light beams. From the studies of spatial coherence on negative and positive GH shifts in the reflected light^29^,^30^, the amplitude of GH shift becomes larger for small values of spatial coherence (*q*) and decreases as the value of the spatial coherence increases. It is remarked that the incident light beam becomes more and more coherent with a larger value of *q* and becomes incoherent with a small value of *q*. In using Eq. (7) to analyze the behavior of GH shift for different number of modes of *p* and *s* polarized partial coherent light, each mode of partial coherent light is perfectly coherent.

**Figure 2:**
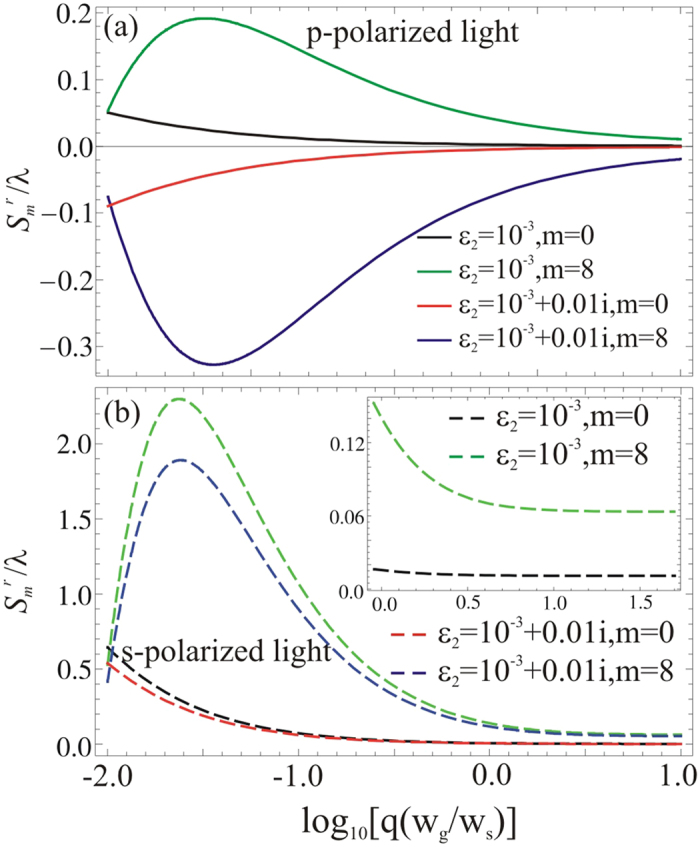
(**a**) The GH shifts versus spatial coherence (*q*) for *p* polarized light. (**b**) The GH shifts versus spatial coherence (*q*) for *s* polarized light, when *w*_*s*_ = 125*λ w*_*s*_ and *θ* = 0.12 rad.

Now, to see the influence of spatial coherence on the GH shift, we start with a *p* polarized light incident on ENZ metamaterials. In Fig. 2(a), we plot the corresponding GH shifts in the reflected light versus spatial coherence *q* for *m* = 0 th- and *m* = 8 th-orders, respectively, with a fixed incident angle *θ* = 0.12 rad. To give a clear representation, first, we concentrate our attention to study the effect of spatial coherence over the GH shift in the reflected light by considering a lossless ENZ metamaterial with the permittivity 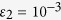. For the lossless ENZ metamaterial, the amplitude of GH shift in the reflected light is high for small values of *q*, and approaches to zero when *q* increases. This clearly shows that the amplitude of the GH shift is not zero for partial coherent light (small *q*) and approaches to zero when light becomes coherent (large *q*), see the black and green curves for different modes of partial coherent light in Fig. 2(a). As reported earlier in ref. 18 that there is no GH shift in the reflected light for *p* polarization because they have used a coherent light beam. This is also shown in our analysis that the GH shift in the reflected light for *p* polarized coherent light approaches to zero as the coherence of the incident beam increases.

Next, we consider the loss effect in the materials, *i.e.*, by setting Im[*ε*_2_ = 0.01]. As shown in Fig. 2(a), the corresponding GH shifts in the reflected light versus the coherence parameter *q* for *p* polarization are shown in the red and blue curves for the *m* = 0 and 8-th modes, respectively. By introducing absorption in a system, the GH shift in the reflected light becomes negative. This is due to the fact that when a *p* polarized light reflected from an absorptive medium, a phase changes abruptly at the Brewester angle and induces a negative GH shift^6^. Similar behavior of the GH shift in the reflected light is also reported for absorptive ENZ metamaterials in ref. 18.

Furthermore, we consider a *s* polarized beam incident on ENZ metamaterials and study the influence of spatial coherence on the GH shift in the reflected light for both lossless and absorptive media. For lossless ENZ metamatrials, positive GH shifts are revealed for a fixed incident angle *θ* = 0.12 rad by considering *m* = 0 and *m* = 8, see the dashed black and green curves in Fig. 2(b). The amplitude of GH shift is larger for small values of spatial coherence and decreases with an increment in the spatial coherence. In the limit of a perfect coherent light field, the amplitude of GH shifts remains as a constant value. To give a clear illustration for such a constant GH shift in the reflected light for coherent light fields, we show the enlarged plot for the GH shifts ranging from 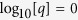 to 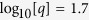 in the inset of Fig. 2(b). The GH shifts in the reflected light in the inset clearly shows that the amplitude remains constant when the value of *q* increases. The corresponding constant amplitude of GH shifts for the coherent light region are 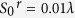
(black curve) and 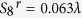
(green curve), respectively. Here, the amplitude of GH shifts in the reflected light is quite small because we consider a large beam width to suppress the distortion of light beam. In contrary to the coherent incident light field, we also reveal that the GH shift in the reflected light does not remain constant for partial coherent light at all the incident angles *θ*. When loss is considered in ENZ metamaterials, a similar behavior on the modified GH shifts in the reflected light can be seen for the dashed red and blue curves in Fig. 2(b).

### Effect of beam widths

**Figure 3:**
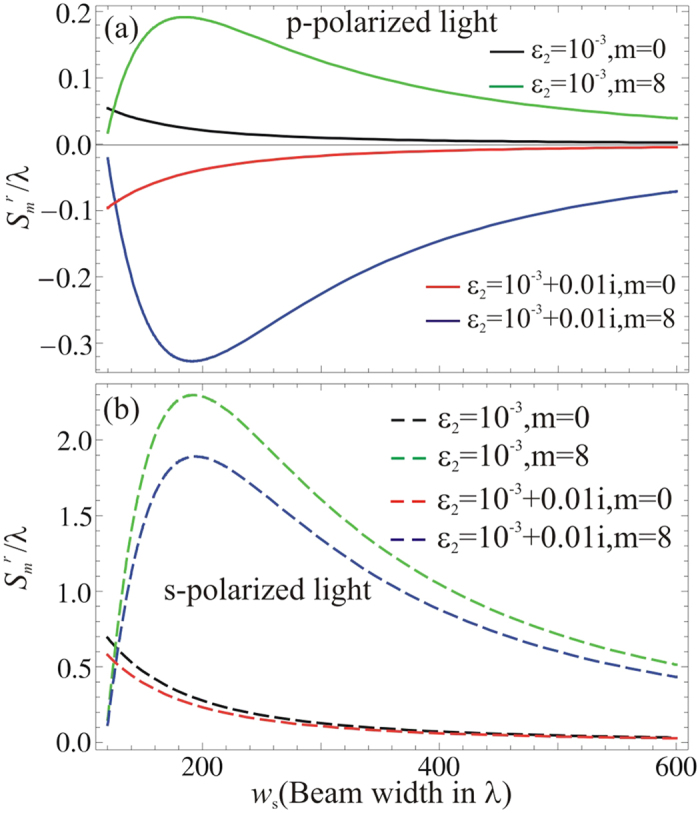
(**a**) The GH shifts versus beam width (*ws*) for *p* polarized light. (**b**) The GH shifts versus beam width (*w*_*s*_) for *s* polarized light, when *q* = 0.01 and *θ* = 0.12 rad.

It is known that the beam width also plays a key role in the observation of GH shifts with a Gaussian beam^22^,^24^, as well as a partial coherent light beam^30^,^31^. By increasing the beam width, the effect of partial coherence on the GH shift decreases and vice versa. Following this concept, here, we consider a spatial coherence *q* = 0.01 for *p* polarized partial coherent light incident on ENZ metamaterials. For lossless ENZ metamaterials, the GH shift in the reflected light for different modes of partial coherent light beam is depicted in Fig. 3(a). The black and green curves in Fig. 3(a) show that the amplitude of GH shift varies with the beam width of partial coherent light, *i.e.*, the amplitude is larger for small values of beam widths and decreases to zero as the beam width increases. Based on these results, one can safely conclude that for small values of beam widths the amplitude of the GH shift is not equal to zero, but approaches to zero for a larger beam width in a *p* polarized light beam.

As an absorptive ENZ metamaterial is considered, in Fig. 3(a). The red and blue curves represent negative GH shifts in the reflected light for *p* polarized light beam, which varies with the beam width, too. These curves of negative GH shifts demonstrate similar behavior as those shown in Fig. 2(a). As for *s* polarized beams, shown in Fig. 3(b), the GH shift in the reflected light is depicted for different modes of partial coherent light field versus beam width *w*_*s*_. Again, we have similar behavior on the GH shift in the reflected light versus the beam width, as those shown in Fig. 2(b) for different spatial coherence *q*. In comparison of Figs 2 and 3, we notice that the influence of spatial coherence *q* and beam width *w*_*s*_ have the same role on the GH shifts in the reflected light of ENZ metamaterials.

### Effect of Re[*ε*_2_]

**Figure 4:**
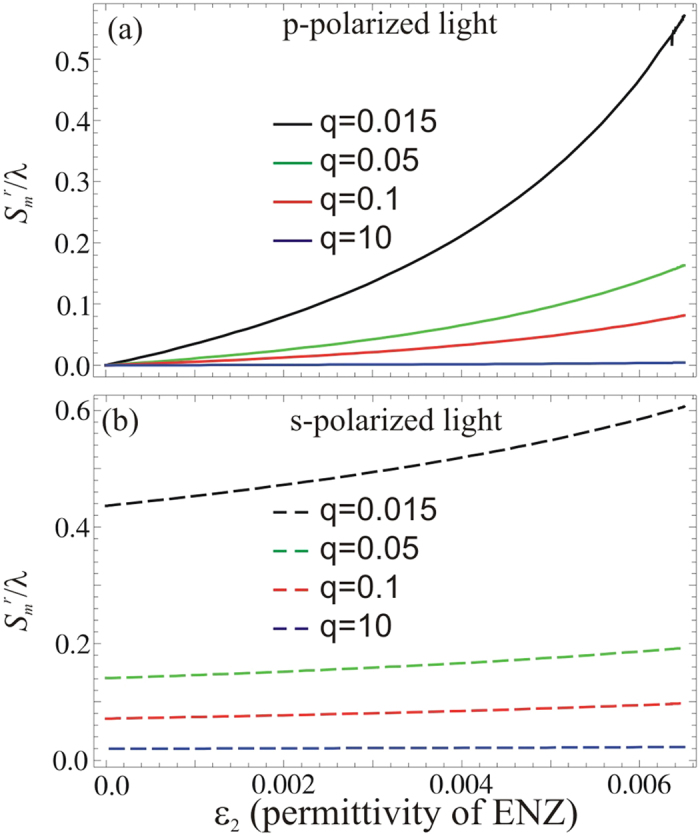
(**a**) The GH shifts versus permittivity of ENZ metamaterials (*ε*_2_) for *p* polarized light. (**b**) The GH shifts versus permittivity of ENZ metamaterials (*ε*_2_) for *s* polarized light, when *m* = 0 and *θ* = 0.12 rad.

By varying the Re[*ε*_2_] (permittivity of metamaterials), it is known that the GH shifts in the reflected light are a little bit affected both for *p* and *s* polarized light^18^. Here, we examine the influence of Re[*ε*_2_] on the GH shift in the reflected light for partially coherent light fields. We consider different values of spatial coherence *q* and study the GH shift for *p* and *s* polarized light, as a function of real part of metamaterial permittivity, see Fig. 4. For different values of *q*, the GH shift in the reflected light for *p* polarized light is shown in Fig. 4(a). There is no GH shift when Re[*ε*_2_] = 0 for different spatial coherence *q*, but for 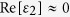 the amplitude of GH shift is non-zero for partial coherent light. Furthermore, the magnitude of the GH shift is large for small values of spatial coherence and decreases as the spatial coherence increases.

The comparison between the black (for *q* = 0.015) and blue (for *q* = 10) curves in Fig. 4(a), clearly shows that the magnitude of GH shift is not zero for partial coherent light (*p* polarized) in ENZ metamaterials. Again, the magnitude of the GH shift approaches to zero in ENZ metamaterials when the light becomes perfectly coherent, *i.e.*, at *q* = 10, see the blue curve in Fig. 4(a). It is also noticed that the magnitude of GH shift for partial coherent light (*p* polarized) increases more quickly with the increment in Re[*ε*_2_], as compared to a coherent light beam (*p* polarized), see the black and blue curves in Fig. 4(a). Similarly, for *s* polarized light beams, as shown in Fig. 4(b), the GH shift is not zero for 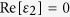 and the magnitude increases for ENZ metamaterials when Re[*ε*_2_] increases. The magnitude of GH shift for *s* polarized partially coherent light is larger than that for a coherent light beam; see the black and blue curves in Fig. 4(b).

## Discussion

In this work, we considered a partial coherent light field incident on ENZ metamaterial with an angle *θ* to the *z*-axis. The corresponding GH shifts in the reflected light both for *p* and *s* polarized beams are studied, with the influence from spatial coherence, beam width, and permittivity of metamaterials. We show that for lossless ENZ metamaterials, the magnitude of GH shift is non-zero for *p* polarized partially coherent light beams; while it approaches to zero as the degree of spatial coherence increases. With small values of spatial coherence and beam width, the GH shifts both for *p* and *s* polarized beams increase first, and then decrease for higher values of spatial coherence and beam width. As in most of practical implementations, such as the x-ray beams^32^,^33^, the coherent light sources are usually not available. With the introduction of partially coherent beams, our results on the GH shifts may provide a direction on the applications for partial coherent light sources in ENZ metamaterials.

## Additional Information

**How to cite this article**: Ziauddin *et al.* Goos-Hänchen shift of partially coherent light fields in epsilon-near-zero metamaterials. *Sci. Rep.* **6**, 26504; doi: 10.1038/srep26504 (2016).
